# Supplementary material for: Paw-spective shift: how our mood alters the way we read dog emotions
Source: PeerJ. 2025 Dec 5;13:e20411. doi: 10.7717/peerj.20411 (PMC12684405; doi:10.7717/peerj.20411)
Supplement: Supplemental Information 2 [file peerj-13-20411-s002.docx]

## Paw-spective Shift: How Our Mood Alters the Way We Read Dog Emotions

Holly G. Molinaro* and Clive D. L. Wynne

Psychology Department, Arizona State University

*Corresponding author: hgmolina@asu.edu

## Supplementary Materials

### NAPS Images Used – Experiment 1

#### Negative Prime


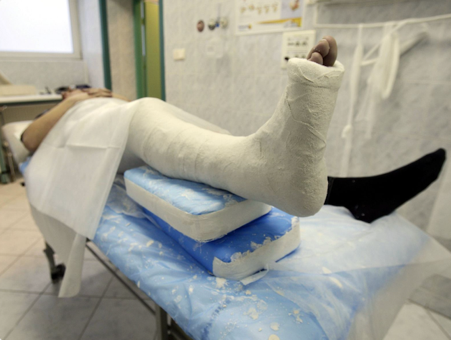


People_210_h


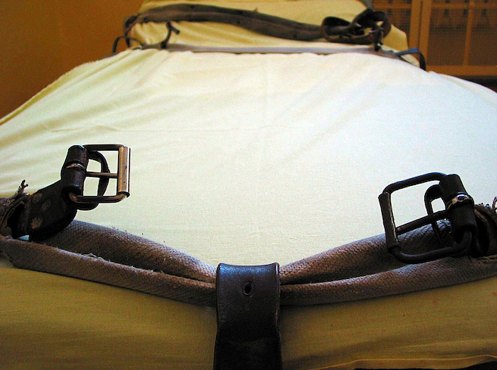


Objects_132_h


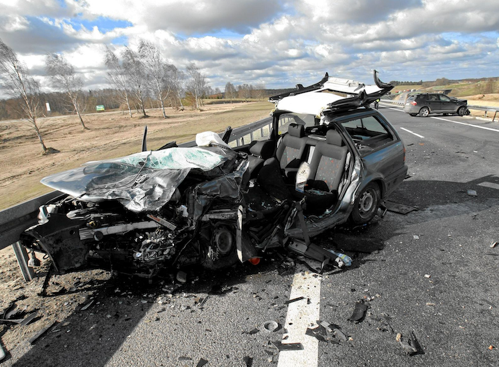


Objects_003_h


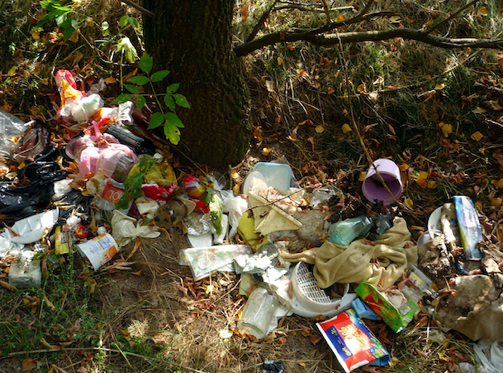


Landscapes_139_h


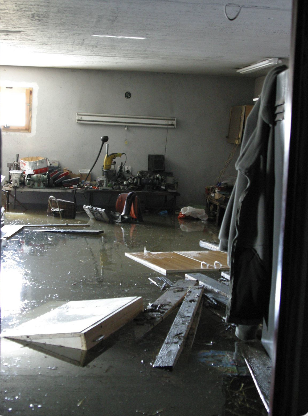


Landscapes_029_v


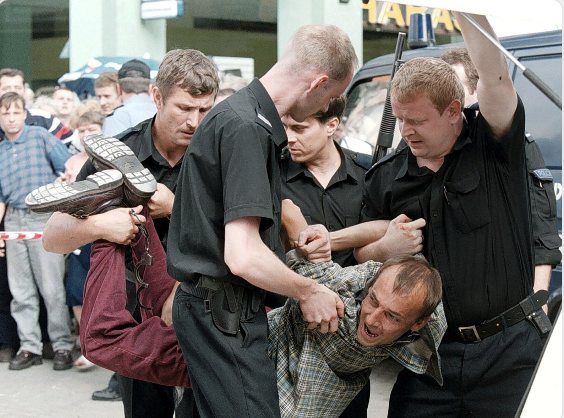


Faces_293_h


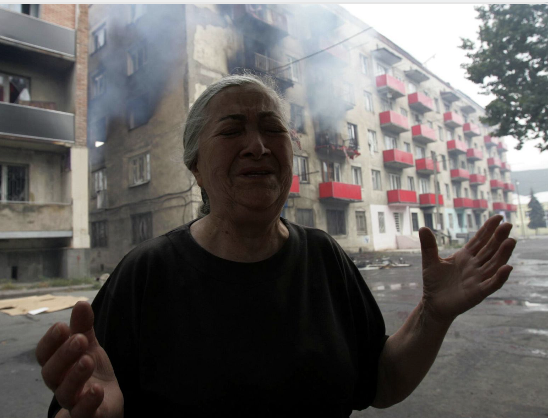


Faces_283_h

#### Neutral Prime


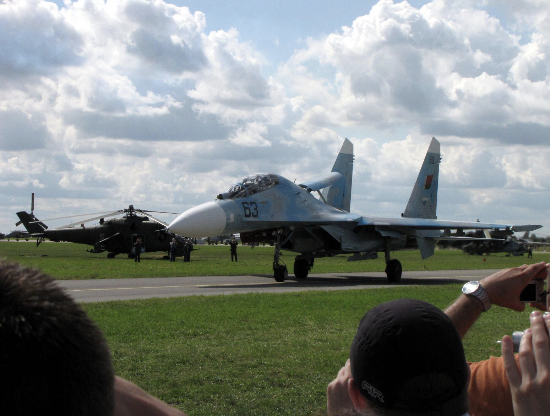


People_248_h


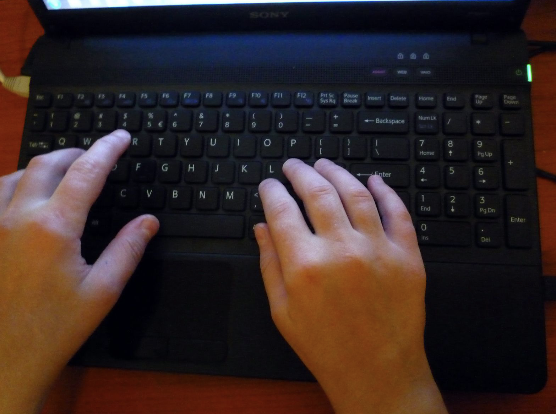


People_091_h


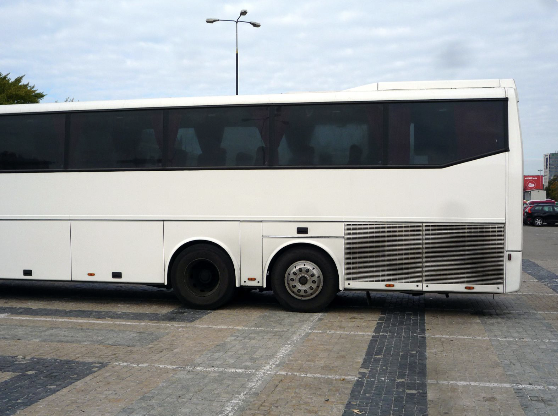


Objects_311_h


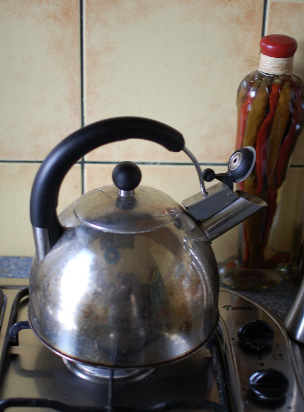


Objects_161_v


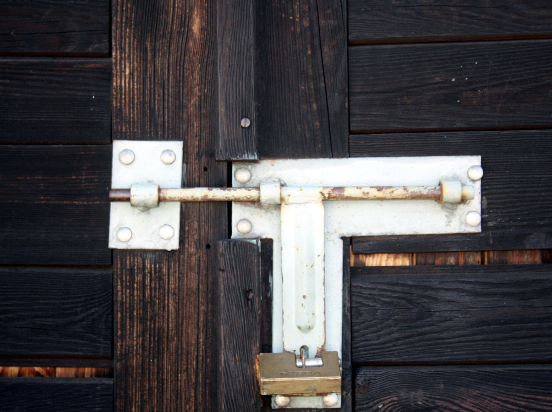


Objects_130_h


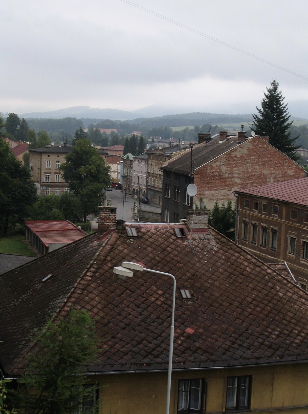


Landscapes_037_v


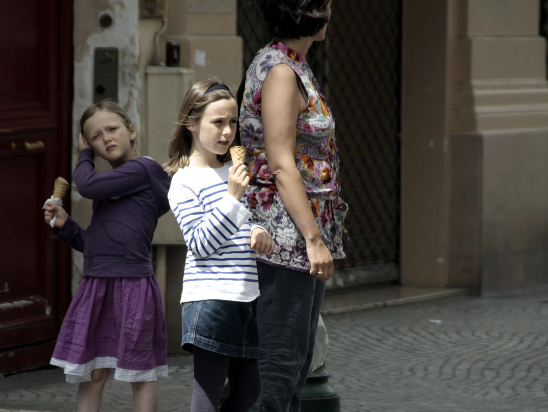


Faces_064_h

#### Positive Prime


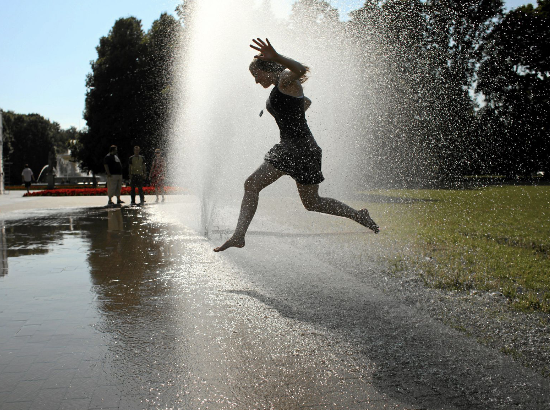


People_187_h


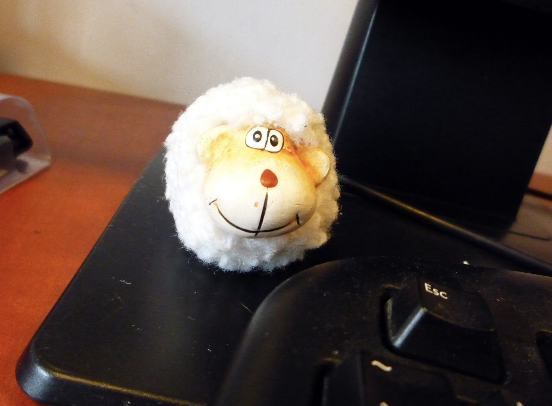


Objects_260_h


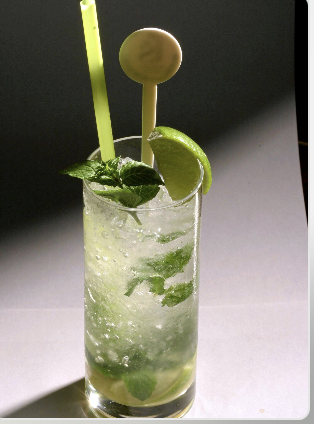


Objects_074_v


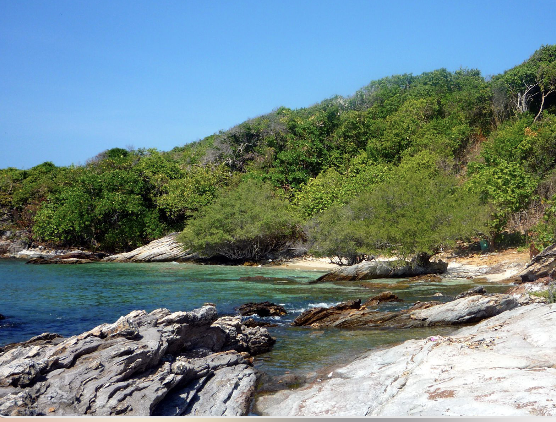


Landscapes_180_h


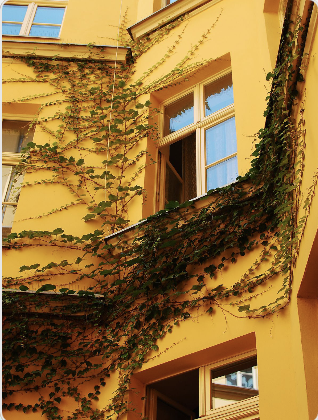


Landscapes_088_v


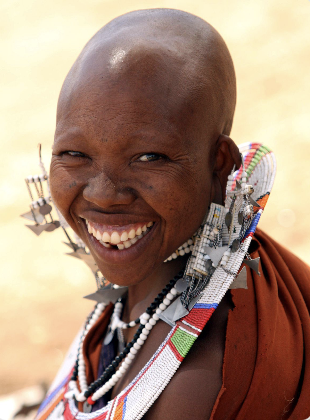


Faces_261_v


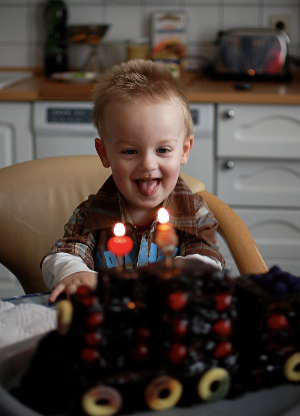


Faces_109_v

### OASIS Images Used – Experiment 2

#### Negative Prime


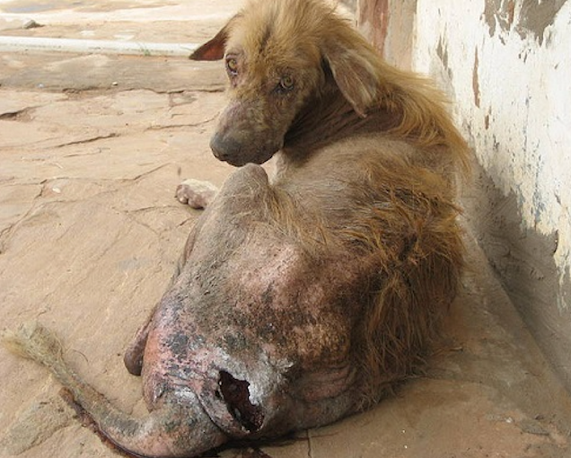


Dog 26


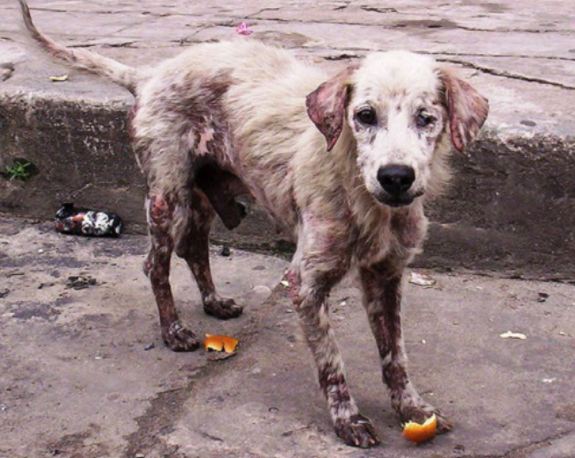


Dog 24


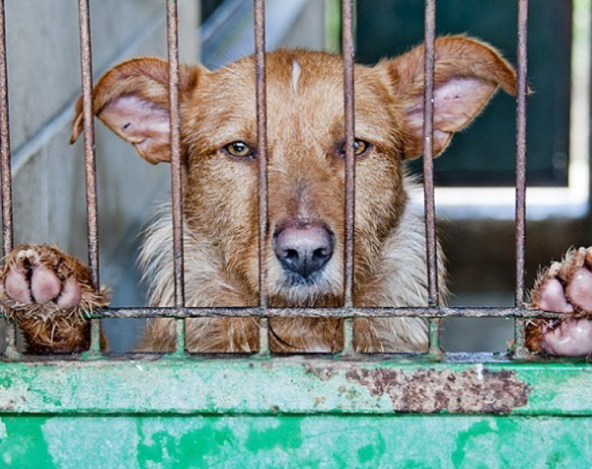


Dog 31


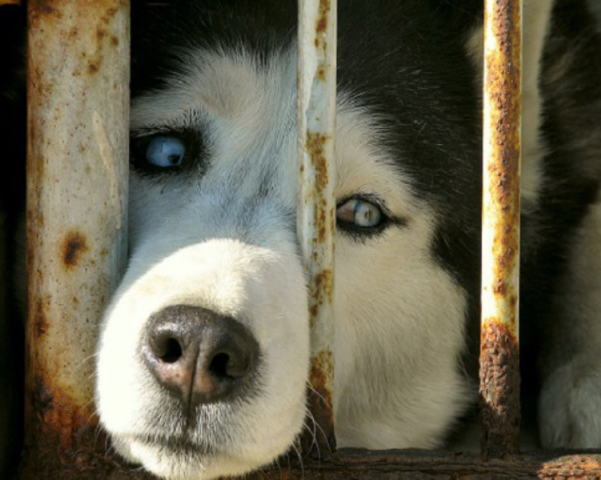


Dog 23


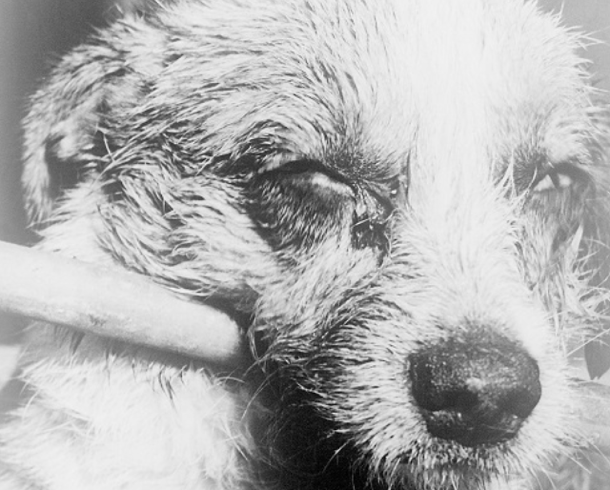


Dog 15


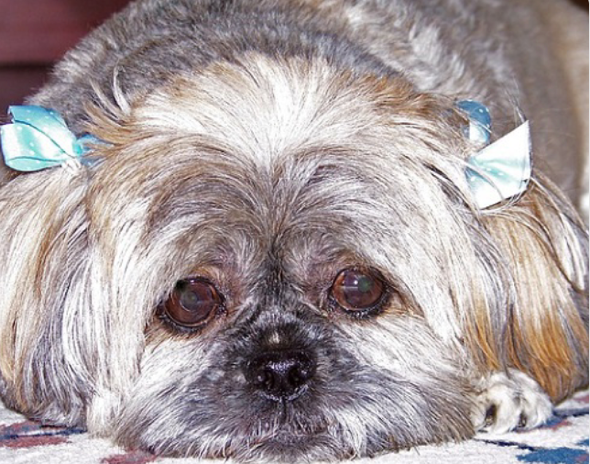


Dog 30


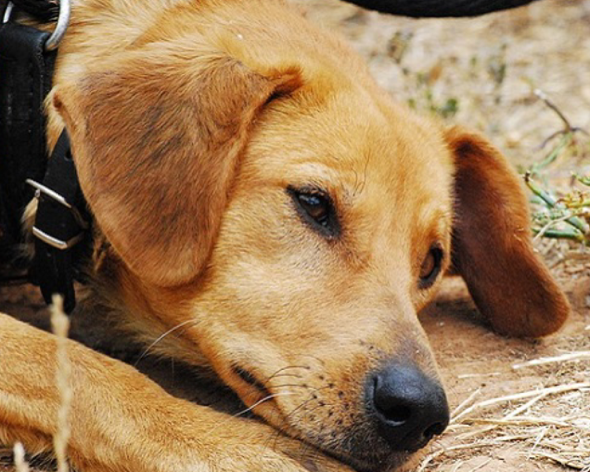


Dog 22

#### Neutral Prime


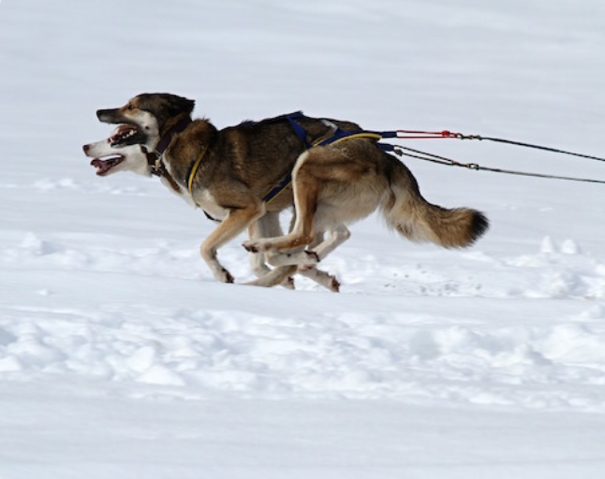


Dog 1


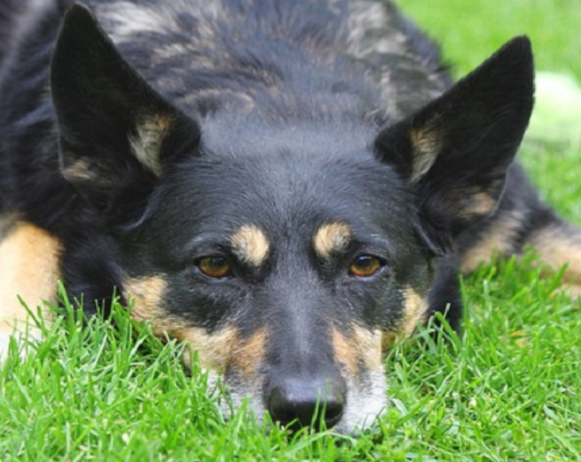


Dog 17


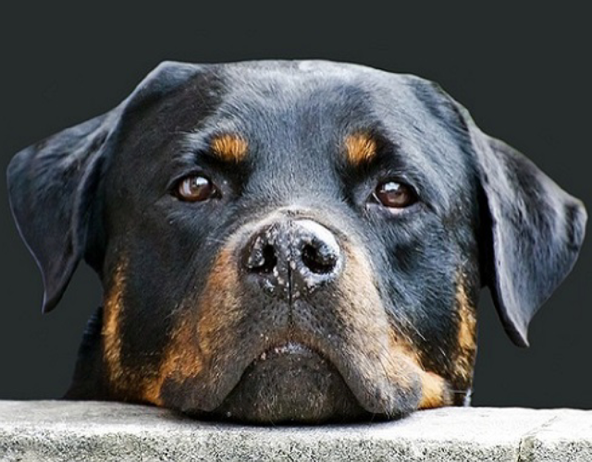


Dog 27


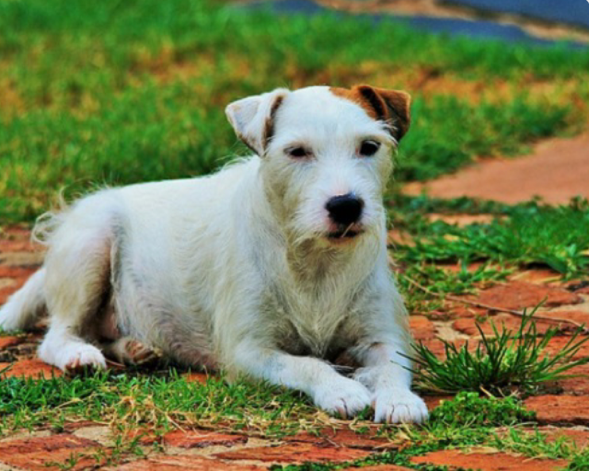


Dog 29


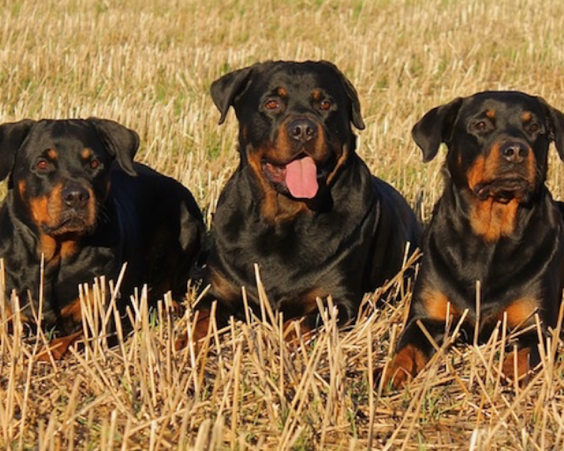


Dog 8


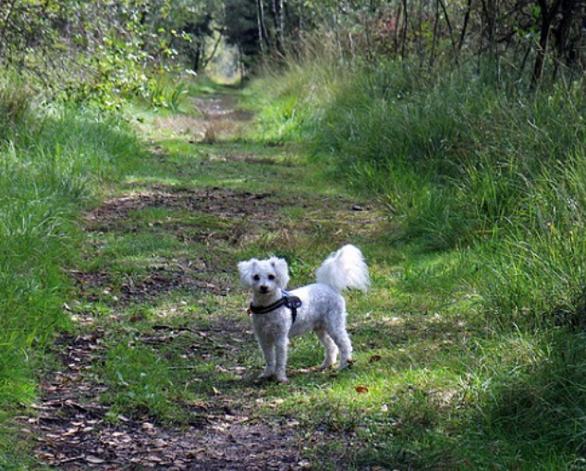


Dog 10


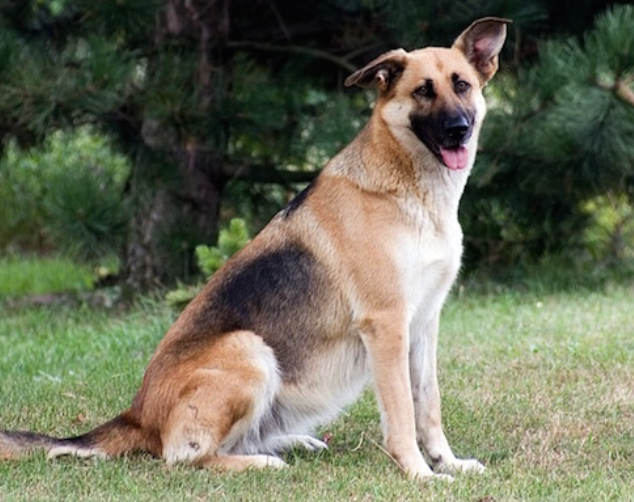


Dog 3

#### Positive Prime


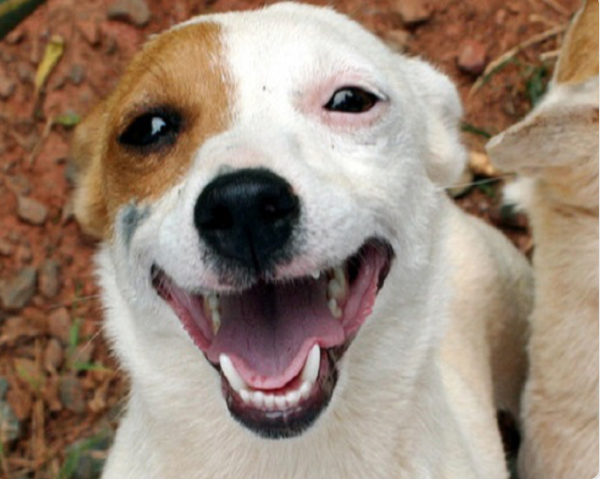


Dog 13


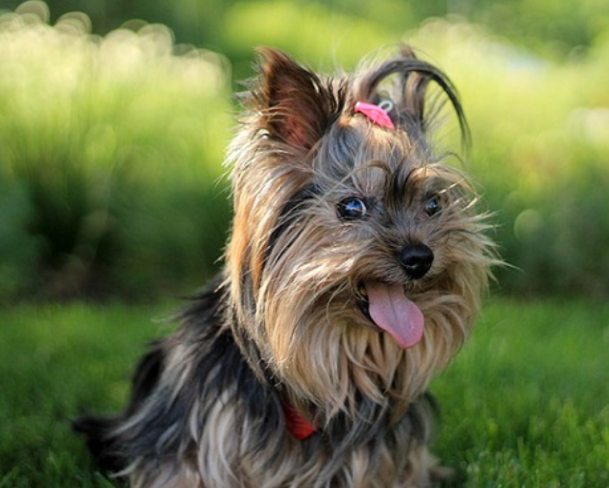


Dog 14


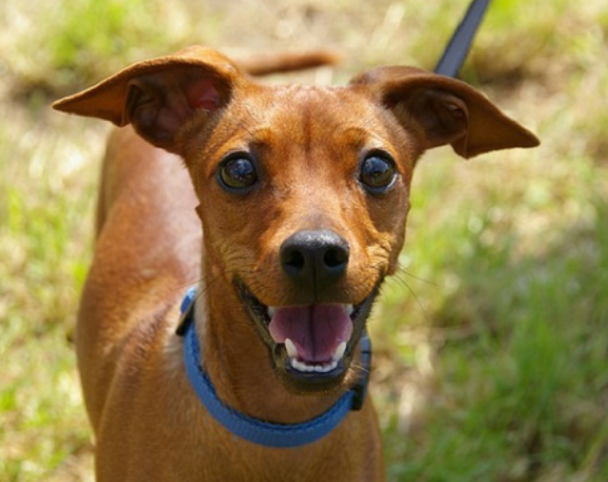


Dog 19


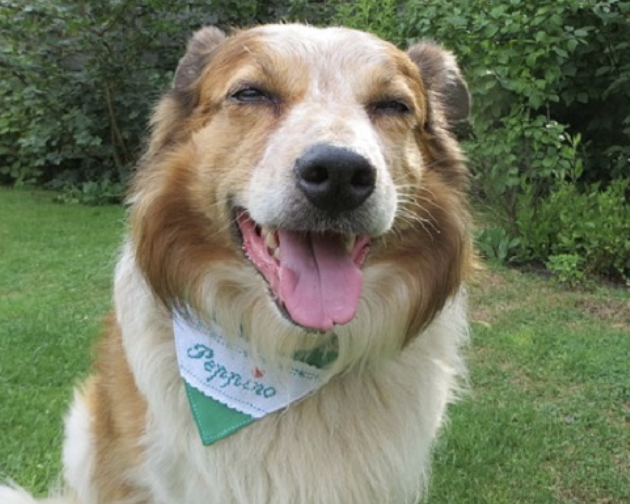


Dog 21


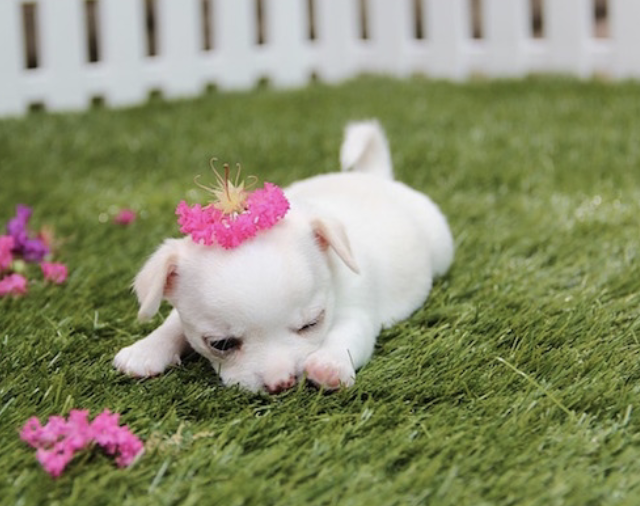


Dog 4


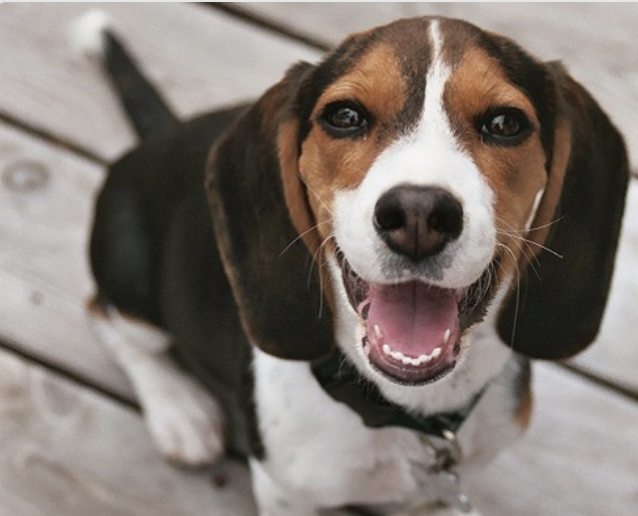


Dog 12


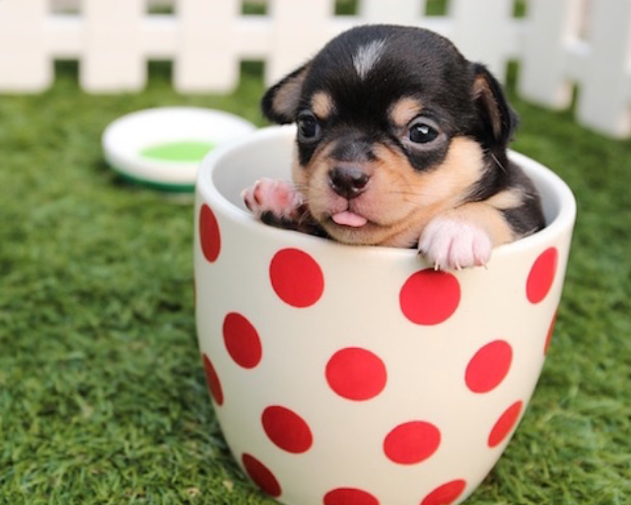


Dog 6

### Videos of Dogs in Experiments 1 and 2

<https://osf.io/fc2wt/>

### Codebook for Analyzing Free Responses

#### Valence (-2,-1,0,1,2)

Valence is the level of positivity or negativity, goodness or badness. We used a scale of 2 to -2 to rate how good or bad a word's meaning is. Negative words fell below zero and were classified according to the intensity of negativity while positive words were classified above anywhere above 0. Words that don’t apply, have indifferent connotations or don’t imply good or bad intentions were rated as 0’s.

**2** = Extremely positive

**1** = Somewhat positive

**0** = Indifferent, passive state for dog, not related to good or bad, could be good or bad depending on perspective, projecting “wanting”, hunger, holds less weight when combined with other levels

**-1** = Somewhat negative

**-2** = Extreme discomfort/negativity

##### Plan of Action:

- If there are two words listed, always go with the “average” (example: 1 and -1 = 0) (2, 2, -1 = 0)
- Always go with the stronger emotion if presented with 2 good traits or 2 bad traits (example: 2 and 1 = 2)
- If there is a neutral value (0), do not include it in the final ranking unless it is the only listed number (example: 2 and 0 = 2)
- If a response gives negative connotation, but you are unsure how bad, go with -1 (or vice versa)
- Two or more of a number will overpower a single number (majority rules)
- If there are multiple conflicting responses (both positive and negative), add up the positive and negative responses and see which one the response is leaning more towards, but do NOT put the strongest emotion (-2 or 2) as the coded one (example: 2, 2, 1, -1, -1 = 1) (2, 2, -1 = 1) (2, -1, -1 = 0)

##### Examples:

           -2         -1   0 1 2

| Aggressive/Angry/Aggravated/Antagonized | Agitated | Active/reactive | Accomplished | Admiration |
| --- | --- | --- | --- | --- |
| Anxious | Aloof | Anticipating/Expectant | Calm/Mellow | Brave |
| Ashamed | Annoyed/Frustration | Aroused | Chillin | Courageous/Bold |
| Belligerent | Awkward | Attentive | Comfortable | Eagerness/Enthusiasm |
| Contempt | Concerned/Startled/Alarmed/Spooked | Aware/Alert | Content | Energetic/Hyper |
| Depressed | Confusion/ Distracted | Bored/Tired/Sleepy | Cool | Engaged |
| Distressed | Dazed | Careless/Carefree | Dominant | Excited |
| Drained | Defensive | Clueless | Flourishing | Love/Affection |
| Dreadful/Miserable | Disinterested | Conscious | Focused | Loyal |
| Frantic | Disoriented | Cooperative | Gentle | Playful |
| Guilty | Fierce | Hungry | Goofy/Silly | Proud |
| Helpless | Flat | Ignorant | Happy | Safe |
| Lonely | Hesitant/Wary/Cautious | Lazy | Hopeful | Secure |
| Neglected | Hot | Looking | Interested | Thrilled |
| Overwhelmed | Impatient | Neutral | Intrigued/Curious |  |
| Paranoid | Impulsive | Neutral Action | Observant |  |
| Pissed | Itchy | Nonchalant/Laid Back/ At Ease | Patient |  |
| Scared/Afraid/Fearful | Lethargic/Sluggish/Fatigue | Numb | Peaceful |  |
| Threatened | Lost | Obedient/Loyal | Pleasant |  |
| Traumatized | Mad | Protective | Relaxed/Resting |  |
|  | Needy/Pesty | Ready to Play or Play | Relief |  |
|  | Reluctant | Serious | Surprised |  |
|  | Restless/Antsy | Stimulated | Trusting |  |
|  | Sad | Suspicion | Welcoming |  |
|  | Sassy | Thinking |  |  |
|  | Shy | Unbothered |  |  |
|  | Skeptical | Unnoticed |  |  |
|  | Stressed | Unwilling |  |  |
|  | Submissive | Waiting |  |  |
|  | Teased |  |  |  |
|  | Territorial |  |  |  |
|  | Unamused/ Unhappy/Upset/Disappointment |  |  |  |
|  | Warning |  |  |  |
|  | Wistful |  |  |  |
|  | Worn out/Exhausted |  |  |  |
|  | Worried/Nervous/Tense/On Edge/Unsettled |  |  |  |

#### Arousal (-2,-1,0,1,2)

Arousal is the degree of alertness or stimulation during the waking state. We used a scale of 2 to -2 to rate the degree of arousal. Words with low arousal are classified below 0 based on the intensity of activity and arousal. Words with high arousal are classified above 0 depending on the intensity of the activity.

**2** = Vocal/talkative, high anxiety/agitation, highly active

**1** = Wanting, trying, happy, playful, interested

**0** = Irrelevant/not applicable, opposites, bare minimum

**-1** = Relaxed but not lethargic

**-2** = Tired, sleepy, very low arousal

##### Plan of Action:

- If there are multiple words listed, always go with the “average” (example: 1 and -1 = 0)
- Always go with the stronger emotion if presented with 2 good traits or 2 bad traits (example: 2 and 1 = 2)
- If there is a neutral value (0), do not include it in the final ranking unless it is the only listed number (example: 2 and 0 = 2)
- If a response gives a low arousal connotation, but you are unsure how low, go with -1 (or vice versa)
- Two or more of a number will overpower a single number (majority rules) (ex: 1, 1, 2 = 1)
- Engaging with the Environment is positive 1 (ex. Looking for something/Smelling Something)
- Take from dog's perspective
- If there are multiple conflicting responses (both positive and negative), add up the positive and negative responses and see which one the response is leaning more towards, but do NOT put the strongest emotion (-2 or 2) as the coded one (example: 2, 2, 1, -1, -1 = 1) (2, 2, -1 = 1)

##### Examples

##### -2     -1    0 1       2

| Calm/Peaceful | Aloof | Awkward | Accomplished | Affection |
| --- | --- | --- | --- | --- |
| Contempt | At Ease | Carefree | Active/Reactive | Antagonized |
| Depressed | Bored | Conscious | Aggravated/Agitated/  Mad | Anxious |
| Drained | Comfortable | Cool | Annoyed | Astonished/Startled/  Spooked |
| Lethargic/Fatigue/  Sluggish | Flat | Flourishing | Aware/Alert/Attentive | Cautious/Hesitant |
| Safe | Laid Back | Hot | Brave/Proud/Goofy/Silly | Defensive |
| Sleepy/Tired | Nonchalant/Chillin/  Chill/Chilling out | Loyal | Concerned/Unsettled | Excited/Energetic |
|  | Numb | Neglected | Contemplating/  Anticipating/Expectant | Focused |
|  | Relaxed/Calming down/relaxing | Neutral | Curious/Confusion/  Skeptical/Suspicious | Frantic/Hyper |
|  | Relief | Obedient | Distracted | Impatient |
|  | Sad | Pleasant | Distressed | Overwhelming/  Consumed |
|  | Shy | Resting | Dominant | Playful |
|  | Submissive | Safe | Engaged/Eager | Restless |
|  | Unamused/Disinterested | Serious | Fierce | Scared |
|  | Unaware | Sitting | Guilty/Ashamed | Starving |
|  | Unbothered | Thinking | Happy/Content/  Enthusiasm | Stressed |
|  | Worn out/Exhausted | Threatened | Hopeful | Terrified/Fearful |
|  |  | Unnoticed | Hungry | Territorial |
|  |  | Waiting | Impulsive | Traumatized |
|  |  |  | Intrigued/Interested/  Observant | Violent/Angry/  Aggressive |
|  |  |  | Itchy |  |
|  |  |  | Lonely |  |
|  |  |  | Looking/Smelling/Seeing |  |
|  |  |  | Lost |  |
|  |  |  | Love |  |
|  |  |  | Patient/Welcoming |  |
|  |  |  | Protective |  |
|  |  |  | Stimulated/Aroused |  |
|  |  |  | Surprised |  |
|  |  |  | Tense/On edge/Wary/Antsy |  |
|  |  |  | Trusting |  |
|  |  |  | Trying to… |  |
|  |  |  | Upset/Disappointed/Frustrated |  |
|  |  |  | Wanting/Needy |  |
|  |  |  | Warning |  |
|  |  |  | Wistful |  |
|  |  |  | Worried/Nervous |  |

#### Anthropomorphism (0,1)

Anthropomorphism refers to the assignment of human characteristics to nonhuman objects or animals. Anthropomorphism encompasses the inference of emotional states from actions and the inference of thoughts. This includes responses such as *”tired,” “happy,”* or *“sad.”* An animal may feel positive or negative emotions but we have no way of knowing whether this is the same as happiness or sadness in humans. Additionally, an animal may lay down but we have no way to know if this is because it is truly tired.

| 0 | 1 |
| --- | --- |
| Active/Reactive | Accomplished |
| Affection | Awkward |
| Aggressive | Brave |
| Attentive | Calm, content/comfortable |
| Awareness/Alert | Carefree |
| Calming down | Conscious |
| Danger | Contempt |
| Distressed | Distracted/Focused |
| Engaged | Drained |
| Flourishing | Fatigue |
| Gentle/Pleasant | Fierce |
| Hot | Flat |
| Itchy | Goofy/Silly |
| Lethargic/Sluggish | Happy/Sad |
| Looking at | Hopeful |
| Negative | Impulsive |
| Neglected | Looking for |
| Neutral | Love |
| Obedient | Loyal |
| Play | No Thoughts |
| Positive | NonChalant/Chillin/Cool/Relaxed |
| Resting | Numb |
| Restless/Hyper | Patient/Bored/Tired |
| Sees something | Playful |
| sitting | Protective/Territorial/Defensive |
| Stimulated | Proud |
| Submissive/Dominant | Right/Wrong |
| Watching/Observant | Scared/Anxious/Frantic |
| Welcoming | Serious |
|  | Shy |
|  | Surprised |
|  | Thinking |
|  | Threatened |
|  | Traumatized |
|  | Trusting |
|  | Unamused |
|  | Unbothered |
|  | Wanting/Curious/Needy |
|  | Warning |
|  | Wistful |

**0=** No anthropomorphic words included in response, observable, objective statements only

**1=** At least one anthropomorphic word included in the response

##### Plan of Action:

- If one anthropomorphism then all thing is anthropomorphism
- If just a verb then no anthropomorphism

#### Mentalizing (0,1)

Mentalizing refers to the assignment of mental attributes or state of mind to a dog. Mental state is something that the human is inferring that the dog might feel. An example of this is *Waiting* vs *Not Moving*. Both are somewhat describing the same thing but *Not Moving* is an observable trait that the dog is exhibiting whereas *Waiting* is inferred. *Waiting* implies the dog is actively thinking about doing or not doing something while Not Moving implies that they are physically not doing something. One must ask themselves can this trait be observed or is it trying to infer what they are feeling / doing.

**1**= Mentalizing, referring to a mental or emotional state

**0**= Not mentalizing, observable actions, behaviors, nothing in box at all

WHY the action is occurring

| 0 | 1 |
| --- | --- |
| Active/Reactive | Accomplished |
| Affection/Needy | Angry/Mad/Upset/Agitated |
| Alert/Attentive | Anxious |
| Antagonizing/Violent/Aggressive | Awkward |
| Any Action (ex: sitting) | Bored/Not having any thoughts |
| Aware | Brave/Proud |
| Calm/Peaceful/Relaxed/Nonchalant/Chillin/At Ease | Carefree |
| Conscious | Comfortable |
| Contemplating | Confusion |
| Cool/Cold | Content/Happy |
| Distracted | Curious |
| Energetic/Hyper/Frantic | Distressed |
| Flat | Drained |
| Gentle/Pleasant | Engaged |
| Goofy/Silly | Fierce |
| Hesitant/Reluctant | Flourishing |
| Itchy | Focused/Looking for… |
| Loyal | Guilty |
| Neglected | Hopeful |
| Neutral | Hot |
| Obedient | Hungry |
| Observant | Impulsive |
| Playful/Friendly/Aloof | Interested/Amused/Intrigued |
| Protective/Defensive | Lethargic/Sluggish/Fatigue |
| Resting | Lonely/Lost |
| Restless/Antsy | Numb |
| Serious (alone) | Sad/Depressed |
| Submissive/Dominant | Scared |
| Teased | Serious (dependant on context) |
| Unnoticed | Shy |
| Waiting (with no other context) | Stimulated |
| Welcoming | Surprised |
|  | Territorial |
|  | Thinking |
|  | Threatened |
|  | Tired/Lazy/Sleepy |
|  | Traumatized |
|  | Trusting |
|  | Unbothered |
|  | Waiting for…/Tired of Waiting |
|  | Wanting… |
|  | Warning |
|  | Wistful |

##### Plan of Action:

- If one response includes mentalizing, then the whole response would be recorded as “present”
- If the word “feels” is present make sure to interpret carefully

#### Action State (0,1)

An action state is when a physical and/ or observable action is performed by the dog itself. If something is being done to the dog or they are feeling an emotion, that does not qualify as an action state. For example, *playful* is an emotion that a dog could be feeling, but for the response to qualify as an observable action state the dog must be physically *playing.* If the response is inferring the dogs intentions behind an action, like being *protective*(subjective) vs the dog simply being *agitated*(objective), it does not qualify as an action state. Any body movement or vocalization, like barking, are considered action states as well.

**1**: Action state, physically observable action, vocalizing

**0**: Not action state, blank, mental state

| 0 | 1 |
| --- | --- |
| Accomplished | Active |
| Aggressive/Hostile | Alert |
| Aware/Observant/Attentive/Alarmed | Anticipating |
| Awkward | Itchy |
| Brave/Proud | Looking at… |
| Concerned/Surprised | Reactive |
| Confused/Contemplating | Relax/Chill |
| Conscious | Relaxing/Chilling/Chillin |
| Defensive/Protective | Resting |
| Distracted/Antsy | Restless |
| Distressed | Sitting |
| Drained | Smelling |
| Energetic/Frantic/Hyper | Territorial |
| Engaged | Trying to (only if there is no mentalizing)… |
| Fierce | Violent |
| Flourishing | Waiting (no other context) |
| Gentle/Carefree/Pleasant/Calm | Warning |
| Guilty/Stressed |  |
| Hopeful |  |
| Hot |  |
| Impulsive |  |
| Lethargic/Sluggish/Fatigue |  |
| Looking/Waiting for… |  |
| Loyal |  |
| Numb |  |
| Obedient/Cooperative/Social |  |
| Playful/Affection |  |
| Relaxed/Calm/NonChalant/Laid back |  |
| Serious |  |
| Shy/Goofy/Silly |  |
| Stimulated/Aroused/Thinking |  |
| Submissive/Dominant |  |
| Thinking |  |
| Threatened |  |
| Tired |  |
| Traumatized |  |
| Trusting |  |
| Unamused |  |
| Unbothered |  |
| Wanting/Desiring/Needy/Aloof |  |
| Welcoming |  |
| Wistful/Neglected |  |

##### Plan of Action:

If any action then 1

If -ing ending then possible action (action IF you are NOT putting yourself in someone’s mind)

If there is a verb, such as “relax,” label it as a 1 since we are unsure if it is relaxing or relaxed

The DOG must be doing the action, not someone else (e.g., the owner)
